# Sites of colonization in hospitalized patients with infections caused by extended-spectrum beta-lactamase organisms: a prospective cohort study

**DOI:** 10.1186/s13756-017-0207-y

**Published:** 2017-05-15

**Authors:** Zeina A. Kanafani, Sukayna M. Fadlallah, Sarah Assaf, Khalil Anouti, Kohar Annie B. Kissoyan, Jad Sfeir, Tamara Nawar, Mohamad Yasmin, Ghassan M. Matar

**Affiliations:** 1 Department of Internal Medicine, American University of Beirut, PO Box 11-0236/11D, Cairo Street, Riad El Solh, Beirut, 1107 2020 Lebanon; 2 Department of Experimental Pathology, Immunology and Microbiology, American University of Beirut, Beirut, Lebanon

**Keywords:** Extended spectrum beta-lactamases, Colonization, Molecular analysis, Antibiotic resistance, Screening

## Abstract

**Background:**

The objective of this study was to determine whether patients infected with extended-spectrum beta-lactamase (ESBL)-producing organisms are colonized at multiple body sites.

**Methods:**

This was a prospective cohort study at a tertiary care center in Beirut, Lebanon. Hospitalized patients with infections caused by ESBL-producing organisms were included. Cultures were obtained from the primary site of infection as well as from other sites (skin, nasopharynx, urine, rectum). Molecular analysis was performed on isolates to determine clonal relatedness.

**Results:**

One hundred patients were included in the study. Only 22 patients had positive cultures from sites other than the primary site of infection. The most common ESBL gene was CTX-M-15 followed by TEM-1. In 11 of 22 patients, isolates collected from the same patient were 100% genetically related, while in the remaining patients, genomic relatedness ranged from 42.9% to 97.1%.

**Conclusions:**

Colonization at sites other than the primary site of infection was not common among our patient population infected with ESBL-producing organisms. The dynamics of transmission of these bacterial strains should be studied in further prospective studies to determine the value of routine active surveillance and the need for expanded precautions in infected and colonized patients.

## Background

ESBL-producing organisms have evolved over the past two decades to become a phenomenon of paramount medical importance at the global level. In the United States, the incidence of ESBL-producing organisms among patients with urinary tract infections rose from 7.8% in 2010 to 18.3% in 2014, and this increase was statistically significant. [1]. Evidence suggests that these organisms have evolved in community-acquired infections [2, 3], and that they are associated with complicated infections [3]. In addition, there exists wide geographical variations in the prevalence of ESBL-producing organisms. In a recent systematic review and meta-analysis, fecal colonization with ESBL-producing organisms was estimated at 14%, with an annual increase of 5.4%, which was mostly pronounced in Africa and Asia (15–46%) compared to Europe (3–6%) and the Americas (2%) [4]. Another study has highlighted the importance of travelers acquiring resistant strains, particularly from destinations in India (72%), South East Asia (59%), and Africa (33%) [5].

Although the problem of ESBL-producing organisms is clearly more accentuated in developing countries [6], it is important to mention that the magnitude of the problem is still probably underestimated due to inadequate or ineffective detection in some clinical laboratories [7, 8]. In the Middle East, reports are available from various countries with growing concerns of emergence of ESBL-producing organisms in the community and hospital settings [9–13]. At AUBMC, the proportion of ESBL-EC and ESBL-KP has risen between 1999 and 2016 from 2.5% and 9.8% to 28.5% and 30%, respectively (data from the Clinical Microbiology Laboratory).

Limiting the spread of ESBL-producing organisms is therefore becoming imperative. Although several studies have looked at antibiotic stewardship as a means of controlling the emergence of multi-drug resistant gram-negative organisms [10, 14, 15], the role of infection control measures is largely unproven and understudied. Infection control interventions have been used mainly in outbreak settings where hospitalized patients are placed under contact isolation precautions [16]. Few reports exist of ESBL-producing organisms that have been isolated from rectal and axillary samples, as well as upper respiratory tract secretions during investigations of outbreaks [16], raising concern for colonization of patients at sites other than those of the primary infection.

Whether this is also true in non-outbreak situations is unclear. Colonization in the absence of outbreaks has so far not been looked at systematically, especially in a high-endemicity area such as Lebanon. In 2009, a study by Friedman et al. showed that 8% of their study subjects had rectal carriage of ESBL-producing organisms at admission to the hospital, and 21% of patients acquired rectal carriage during their hospital stay [17]. However, it remains to be determined whether additional colonization sites exist, and whether the organisms at colonization sites are identical by molecular methods to those at the primary site of infection.

The objective of this study is to evaluate the extent of colonization with ESBL-EC and ESBL-KP in hospitalized patients with active infections and designing recommendations accordingly. The results of this study will have implications on infection control practices and will constitute a prerequisite for future studies.

## Methods

### Study design and patient population

This was a prospective cohort study conducted at AUBMC, a 350-bed tertiary care center, and one of the major referral hospitals in Lebanon and the region. Adult patients who were hospitalized at AUBMC between July 2011 and February 2014 and who were diagnosed with an infection due to ESBL-EC or ESBL-KP were included in the study. Patients were identified through the Clinical Microbiology Laboratory based on culture specimens growing ESBL-EC or ESBL-KP. Each patient was included in the study only once, based on the first culture result within the period of the study. Patients whose cultures were deemed to represent colonization rather than true infection were excluded. In addition, patients were excluded if they had infection with the same antibiotic-resistant organism within the preceding year and if they had been on effective antibiotic therapy for longer than 48 h at the time of enrollment.

Patient-specific clinical and laboratory data were collected prospectively from patients’ medical records, including demographics, comorbidities, information about the current infection episode, recent exposure to the healthcare system, and recent antibiotic intake.

### Study intervention

Screening cultures for potential sites of colonization were performed on each patient upon enrollment in the study. Culture specimens were obtained from the skin (axillary, umbilical, and inguinal areas), nasopharynx, urine, rectum, and wounds (if applicable).

### Microbiological analysis

Clinical isolates of ESBL-EC and ESBL-KP that are recovered from different specimens submitted to the Clinical Microbiology Laboratory of AUBMC were analyzed. Specimens on swabs were inoculated in Trypticase soy broth overnight, then subcultured on MacConkey agar and blood agar plates. Colonial morphology on both types of plates were visualized and identified presumptively based on colonial morphology and Gram staining. Identification at the species levels was done by inoculation in API 20E and deciphering the species type using a software provided by the manufacturers. Susceptibility testing against relevant antimicrobial agents was performed using the Mueller-Hinton agar disk diffusion method according to the Clinical Laboratory Standards Institute (CLSI) guidelines. ESBL production was suspected based on susceptibility of the isolate to cefoxitin and imipenem and intermediate susceptibility or resistance to aztreonam, cefotaxime, and/or ceftazidime. The confirmation of ESBL production was conducted by testing the following antibiotic disks: cefotaxime (30 μg), cefotaxime/clavulanate (30/10 μg), ceftazidime (30 μg), and ceftazidime/clavulanate (30/10 μg) on Mueller–Hinton agar according to the CLSI guidelines.

### Molecular analysis

ESBL producing isolates were received, cultured on MacConkey agar (Scharlau, Spain) and stored in Brucella broth (BBL, enriched with 15% glycerol) at -80C for later use.

DNA was extracted from the isolates using the InstaGene Matrix (BIO-RAD, CA), following the manufacturer’s instructions for DNA preparation from bacteria. Polymerase chain reaction (PCR) for CTXM-15 and TEM-1 were performed on the extracted DNA [18]. The gene CTXM-15 was amplified using forward primer 5′-GCGTGATACCAC TTCACCTC-3′ and reverse primer 5′-TGAAGTAAGTGACC AGA TC-3′, and the gene TEM-1 was amplified using forward primer 5′-ATGAGTATTCAA CATTTCCG-3′ reverse primer 5′-CCAATGCTTAATCAGTGAGG-3′ (Thermo Fisher Scientific, Germany). Amplification was achieved using the PCR Sprint Thermal Cycler (Thermo Fisher Scientific, Waltham, MA, USA). Cycling conditions were previously optimized in house for both genes, with an annealing temperature of 50 °C. PCR amplicons were electrophoresed on 1.5% agarose gel using SeaKem® LE Agarose (FMC BioProduct, Rockland, ME, USA). Amplicons were then observed using ULTRA LUM, Dual Light Transilluminator (Claremont, CA), and photographed using a digital camera (Olympus). A permanent record was saved using the Digi-Doc It program (Ultra Violet Products Ltd., Cambridge, UK).

Pulsed-field gel electrophoresis (PFGE) was carried in the CHEF MAPPER (BIORAD, Austin, Texas, USA). The DNA was digested using *Xba*I enzyme (Thermo Fisher Scientific, Waltham, MA, USA) on *Escherichia coli* and *Klebseilla pneumoniae* to determine their genomic relatedness using the standard operating procedure for PulseNet PFGE of *E. coli* O157:H7, *E. coli* nonO157 (STEC), *Salmonella* serotypes, *Shigella sonnei*, and *Shigella flexneri* [19]. DNA patterns were visualized as described above and a dendrogram showing percent DNA relatedness among tested isolates was generated using the BIONUMERCS software (Applied Math, ULM, Germany).

### Ethical considerations

The study was approved by the Institutional Review Board at the American University of Beirut. Informed consent was obtained from patients or their legal representative prior to enrolment. Access to patient-related data was restricted to study personnel and all patient identifiers were removed from the final dataset.

### Data analysis

Data were entered into an electronic database using IBM SPSS Statistics Version 21. Bivariable analysis was used to detect statistical associations. The chi-square test and the independent samples t-test were used for categorical and continuous variables, respectively. The level of significance was drawn at *p* < 0.05.

## Results

A total of 100 patients were enrolled in the study. Clinical data were available on 85 patients (15 patients gave consent to obtain cultures but did not consent to access to their medical records). The baseline characteristics of these 85 patients are represented in Table 1. The patient population was relatively old, with a mean age of 66.9 years. The most common underlying comorbidity was malignancy (48.2%) followed by diabetes (32.9%). As for recent exposure to healthcare, 41.2% of patients had been admitted to the hospital, 18.8% had urinary catheter placement, and 21.2% of patients had undergone a surgical procedure within 30 days of the current infection. In addition, a significant proportion of patients had received antibiotics recently.

**Table 1:** Baseline characteristics of 85 enrolled patients on whom clinical information was available

Variable	Value^a^ (*N* = 85)
Age in years, mean ± standard deviation	66.9 ± 15.9
Male gender	33 (38.8)
Comorbid conditions	
Malignancy	41 (48.2)
Diabetes mellitus	28 (32.9)
Corticosteroid/Immunosuppressive therapy	22 (25.9)
Renal insufficiency	11 (12.9)
Chronic pulmonary disease	8 (9.4)
Exposure to healthcare/invasive devices within 30 days of current infection	
Hospital stay	
0–2 days	50 (58.8)
3–5 days	12 (14.1)
> 6 days	23 (27.1)
Urinary catheter	
0–2 days	69 (81.2)
3–5 days	7 (8.2)
> 6 days	9 (10.6)
Surgical procedure	18 (21.2)
Recent antibiotic use	
Within 24 h prior to enrollment	15 (17.6)
For >48 h within 30 days prior to enrollment	32 (37.6)
Place of acquisition of infection	
Community-acquired	37 (43.5)
Healthcare-associated	29 (34.2)
Hospital-acquired	19 (22.3)
Site of infection	
Urinary tract	74 (87.1)
Skin and soft tissue	6 (7.1)
Bloodstream	4 (4.7)
Respiratory tract	1 (1.2)

^a^Numbers represent *n* (%) unless otherwise indicated

**Fig. 1:**
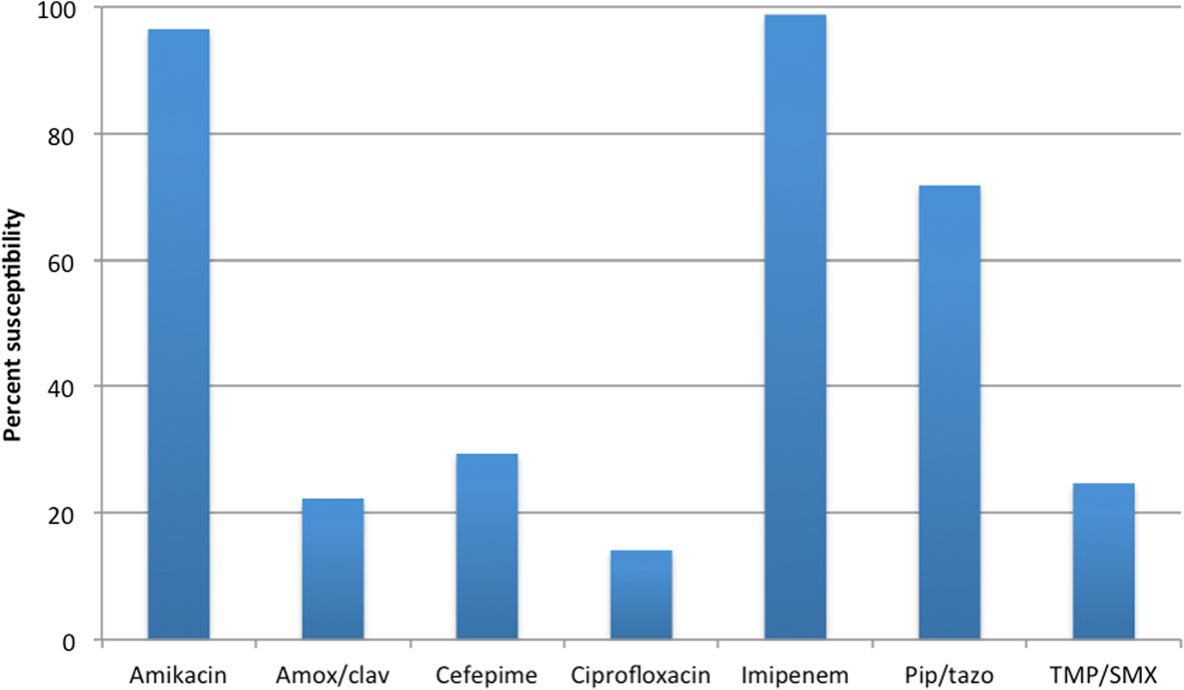
Susceptibility pattern of ESBL-producing Enterobacteriaceae isolates to various antimicrobial agents. Amox/clav = amoxicillin/clavulanate; Pip/tazo = piperacillin/tazobactam; TMP/SMX = trimethoprim/sulfamethoxazole

An excess of 40% of episodes of infections were classified as community acquired, and the urinary tract was the most frequent source of infection. Of all infections, 87.1% were caused by *E. coli* and 12.9% by *Klebsiella* spp. Susceptibility testing revealed high susceptibility rates of the isolates to amikacin (96.5%), carbapenems (98.8%), and piperacillin/tazobactam (71.8%) (Fig. 1). Cefepime, fluoroquinolones, and trimethoprim/sulfamethoxazole performed poorly.

**Fig. 2:**
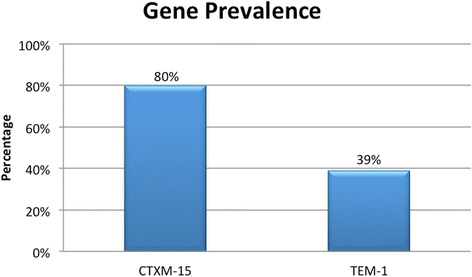
Gene prevalence of CTX-M-15 and TEM-1 in 54 isolates

Only 22/100 patients (22%) had positive cultures at sites other than the original source of infection, i.e. were colonized with ESBL-producing organisms. Isolates recovered from various screening sites in these 22 patients were subjected to molecular analysis (total of 54 isolates). PCR results showed that 80% of the isolates tested were positive for CTX-M-15, while 39% were positive for TEM-1 (Fig. 2). While in some patients (*n* = 11) the same genes were detected from the different isolates collected from various sites, in other patients (*n* = 10), there were minor variations in the genetic distribution in the isolates recovered from different sites (Table 2).

**Table 2:** PCR results for the two tested genes CTX-M-15 and TEM-1 in the isolates tested

Patient Number	Isolate	CTX-M15	TEM-1	PFGE
1	1	Pos	Pos	No PFGE
1	2	Pos	Pos	No PFGE
1	3	Pos	Pos	93.3%
1	4	Pos	Pos	
1	5	Pos	Pos	
1	6	No Growth		
2	1	Pos	Neg	100%
2	2	Pos	Neg	
3	1	No Growth		97.1%
3	2	No Growth		
4	1	Pos	Neg	100%
4	2	Pos	Neg	
5	1	Pos	Neg	100%
5	2	Pos	Neg	
6	1	Pos	Neg	100%
6	2	Pos	Neg	
7	1	Pos	Neg	100%
7	2	Neg	Neg	
8	1	Pos	Neg	100%
8	2	Pos	Neg	
8	3	Pos	Neg	
9	1	Pos	Pos	55.3%
9	2	No Growth		
9	3	Pos	Pos	
9	4	Pos	Pos	
9	5	Pos	Pos	
9	6	Pos	Pos	
10	1	Pos	Pos	60.3%
10	2	Pos	Neg	
10	3	Neg	Pos	
10	4	Neg	Pos	
11	1	Pos	Pos	59.8%
11	2	Pos	Pos	
11	3	Neg	Neg	
12	1	Pos	Neg	60%
12	2	Neg	Neg	
13	1	Neg	Neg	60.9%
13	2	Neg	Neg	
14	1	Pos	Neg	42.9%
14	2	Pos	Pos	
15	1	Pos	Pos	100%
15	2	Neg	Neg	
15	3	Neg	Neg	
16	1	Pos	Neg	97.1%
16	2	Pos	Neg	
16	3	Pos	Neg	
17	1	Pos	Neg	100%
17	2	Pos	Neg	
18	1	Pos	Pos	100%
18	2	Neg	Pos	
19	1	Pos	Neg	100%
19	2	Pos	Neg	
20	1	Pos	Neg	100%
20	2	Pos	Pos	
21	1	Pos	Neg	56.6%
21	2	Pos	Pos	
22	1	Pos	Neg	63.4%
22	2	Neg	Neg	

*PCR* polymerase chain reaction, *PFGE* pulsed-field gel electrophoresis, *Pos* positive, *Neg* negative

**Fig. 3:**
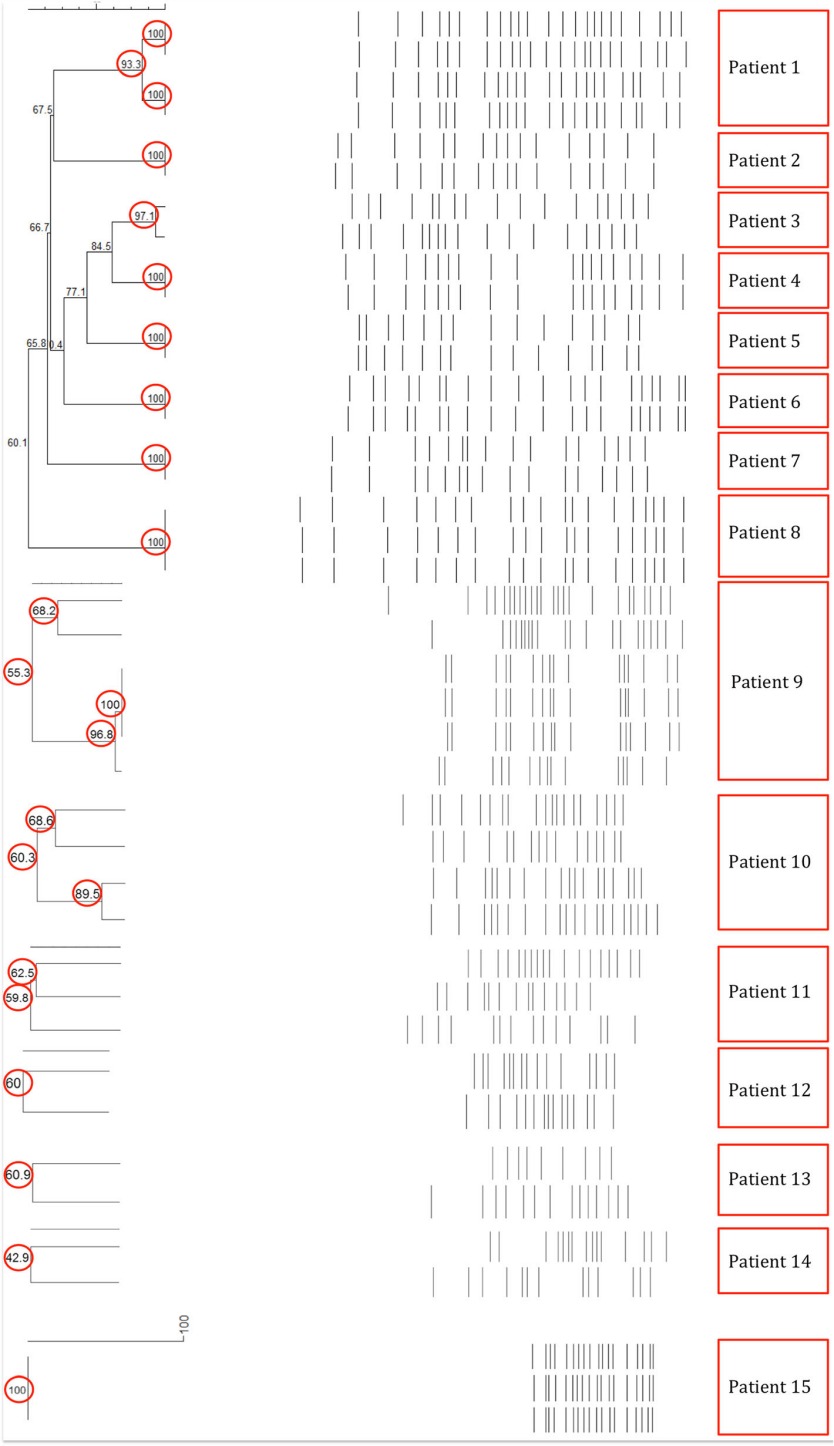
Genomic relatedness between *E. coli* isolates

**Fig. 4:**
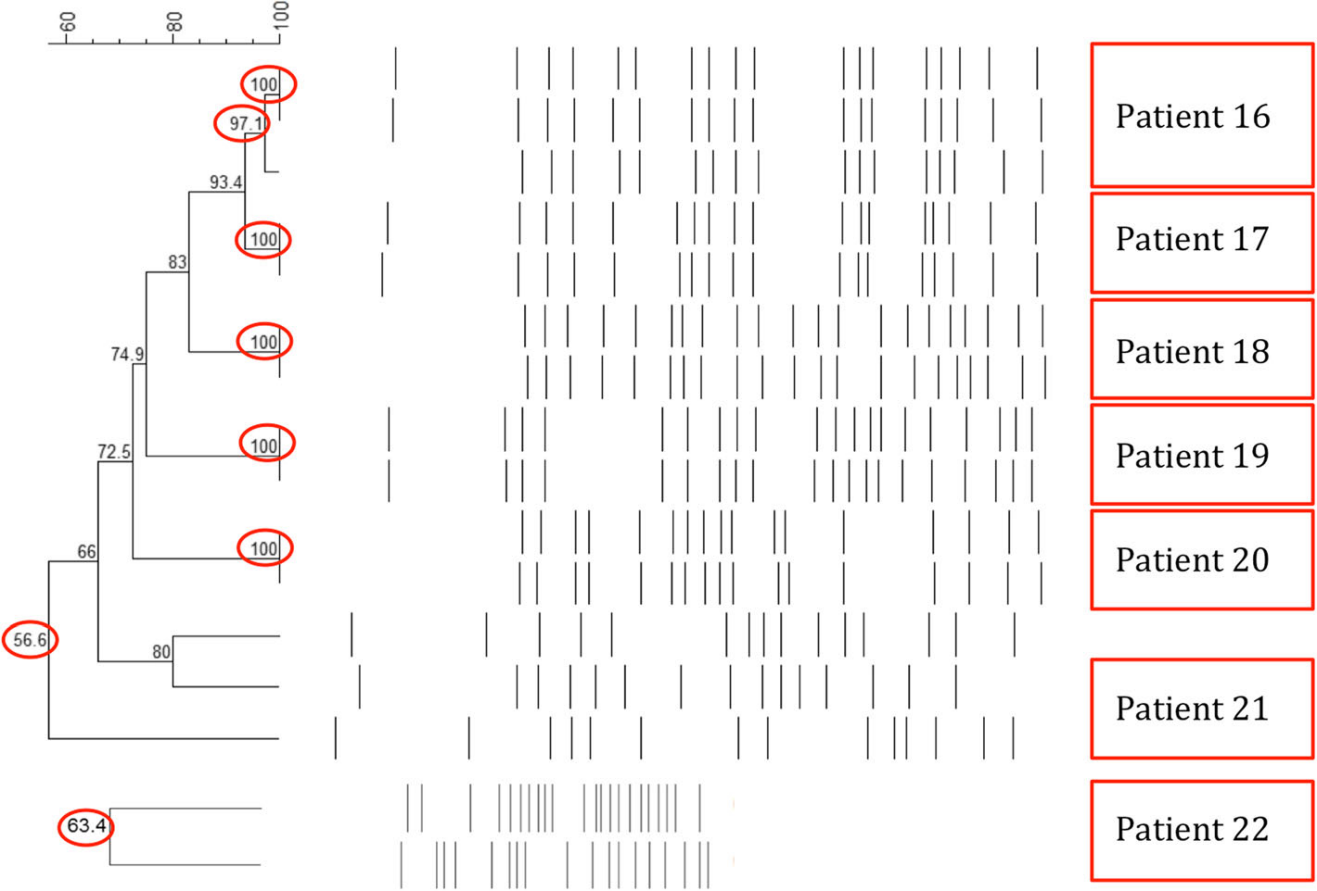
Genomic relatedness between *K. pneumoniae* isolates

The PFGE analysis indicates that isolates collected from the same patient were 100% genetically related in 11 of the 22 patients, while in the rest of the patients genomic relatedness varied between 42.9% and 97.1% (Figs. 3 and 4). To note is that there were two isolates with no PFGE data, and four isolates with no PCR data, since the bacteria did not grow at the time of the performed experiments.

There were no significant correlations between any of the patient factors and the results of molecular analysis. In particular, place of acquisition of infection was not associated with any specific ESBL gene, or with a specific PFGE pattern.

## Discussion

The rising incidence of infections caused by extended-spectrum beta-lactamase (ESBL) producing Enterobacteriaceae is of worldwide dimensions, particularly in developing countries. A recent review in the Lancet describes the epidemiology of ESBLs as “more complex with increasingly blurred boundaries between hospitals and the community” with a trend towards the emergence of community-onset bloodstream infections, making ESBL producing Enterobacteriaceae an “emerging public health concern” [9]. In the present study, around 40% of infections were community-acquired, which has important implications for the initiation of empiric antibiotic therapy in patients with community-onset infections.

The most frequent underlying disease in our patient population was malignancy (48.2%), followed by diabetes mellitus 32.9%). These rates are higher than those seen previously at our center [20], and could be accounted for by the older age of the current patient population (mean age 67 years in this study compared to 47 years in the previous study).

Colonization at sites other than the primary source of infection was detected in 22% of cases only, half of which consisting of identical strains. Active surveillance for ESBL-producing organisms is not routinely performed at AUBMC, i.e. baseline cultures are not obtained upon hospital admission, except in the intensive care and respiratory care units. Therefore, in current practice, clinicians make decisions about isolation precautions after initial culture results become available rather than upon admission and prior to antibiotic initiation. The design of this study therefore mirrors this practice by assessing the presence of colonization around 48 h after initial cultures. It may be assumed that some patients would be colonized at baseline but that secondary cultures would be negative due to empirical antibiotic coverage. However, in such cases, the risk of transmission of the organism would be deemed low, and therefore isolation precautions would not be necessary. Patients who had been on effective antibiotics longer than 48 h prior to the time of obtaining secondary cultures were excluded from the present study.

The current policy at AUBMC does not mandate contact precautions for patients with infections caused by ESBL-producing organisms. This infection control guidance is based on consensus rather than on published evidence.

Our initial hypothesis was that patients with infections due to ESBL-producing organisms would be colonized at body sites other than the primary site of infection, therefore constituting a risk of transmission of the organism from these sites of colonization to the patient environment and the hands of healthcare workers. Although we found that the colonization burden is low among infected patients, we did not assess those patients who are only colonized, and who may very well become sources of infection for others. The lack of active surveillance therefore limits our ability to construct a complete picture about the dynamics of transmission of ESBL-EC and ESBL-KP in the hospital. Another factor to be taken into consideration is that a large proportion of infections were deemed to be hospital-acquired. Whether these infections were the result of direct transmission from another infected or colonized patient, or whether they emerged de novo under the pressure of antibiotic therapy is to be determined and requires further studies.

The two ESBL genes most frequently detected in the tested isolates in this study were CTX-M-15 (80%), and TEM-1 (39%). This is in congruence with previous data from our center that showed CTX-M-15 to be the most common ESBL enzyme produced among *E. coli* and *Klebsiella* species [18]. High prevalence of the CTX-M gene has been described in various reports from different geographical regions including Europe [21], Asia [22], and the Americas [23]. Of interest is evidence suggesting that this enzyme is expressed in bacterial strains harbored by dogs, livestock, and birds [24, 25].

The results of PFGE analysis showed genomic relatedness among isolates recovered from the same patients that ranged from 42.9% to 100%. In fact, some patients were found to be colonized with multiple genetically distinct strains. These findings speak against the endemicity of a single ESBL-producing bacterial strain at our institution, and are consistent with our previous work [18]. Polyclonal dissemination of Gram-negative multi-drug resistant strains has been previously documented in multiple studies [26]. In a recent review, Calbo and Garau confirm that with the emergence of CTX-M-15 production, plasmids with multiple ESBL genes have been described, and polyclonal outbreaks have become more common [27].

## Conclusions

We found that a minority of patients with ESBL-EC and ESBL-KP infections are colonized at body sites other than the primary site of infection. CTX-M-15 is the predominant ESBL gene harbored by these strains, and multiple bacterial clones are circulating at our institution. Further prospective surveillance studies are needed to evaluate the need for expanded precautions in hospitals where ESBL-EC and ESBL-KP are considered endemic. Other infection control measures such as antibiotic stewardship should also be implemented to curb the spread of ESBL-producing organisms.
